# Postharvest Handling Practices and Perception of Potato Safety among Potato Traders in Nairobi, Kenya

**DOI:** 10.1155/2019/2342619

**Published:** 2019-04-28

**Authors:** Consolata Nolega Musita, Michael Wandayi Okoth, George Ooko Abong'

**Affiliations:** Department of Food Science, Nutrition and Technology, University of Nairobi, Kenya

## Abstract

Postharvest handling of the potato is an important factor not only in preventing postharvest losses but also in maintaining its safety and nutritional quality. Exposure of the potato to unfavorable conditions such as light, extreme temperatures, and bruising can result in accumulation of glycoalkaloids, which are toxic substances. This study was a cross-sectional survey which aimed to investigate the postharvest handling practices of potatoes and perception of potato safety among open air market traders in Nairobi County, Kenya. Information was collected from 100 potato traders using a semistructured questionnaire that assessed postharvest handling practices such as potato transportation, exposure to sunlight, and storage. Results indicated that most of the potatoes (88%) took one day to be transported to the market, with the storage period at the market ranging from 2 to 3 days for most traders (42%). Forty-seven percent (47%) of the vehicles and hand-pulled carts used to transport potatoes had open backs, while 53% had closed backs. Over half (69%) of the potatoes in the markets were directly exposed to sunlight, with 75% of the traders leaving their potatoes in the open covered with a polythene bag after the day's activities. Greening, sprouting, or bruised potatoes were mostly sold as seed, sold to restaurants and French fries vendors, or sold to consumers at a lower price. More than half of the traders did not think that consumption of greened potatoes is harmful to health. The results clearly show that there is poor handling of the potatoes by the traders which increases the risk of consumer exposure to glycoalkaloids. There is, therefore, a need to create awareness among traders on appropriate postharvest handling of potatoes to protect consumer health and reduce economic losses as well.

## 1. Introduction

The potato (*Solanum tuberosum) *is an important staple food crop globally. The crop is ranked third after rice and wheat in terms of global human consumption with more than 1 billion people eating it regularly [1]. It is a crop with stable yields and high nutritional content and is, therefore, important in terms of achieving food and nutritional security. In addition to being a nutritious staple food crop, it is an important income earner for all the players along its value chain from production, marketing, and processing.

In Kenya, potato production and consumption are on the increase with the crop being second to maize in terms of consumption. In 2010, Kenya's annual potato production averaged 7.7 tonnes per hectare [2] with the main potato growing regions being Nyandarua, Bomet, Meru, Nakuru, Uasin Gishu, Narok, Nyeri, Kiambu, West Pokot, and Keiyo Marakwett [3]. There are 19 adapted potato varieties in Kenya [4] with Shangi variety being the most common and highly consumed variety due to its early maturity and high productivity although the number of varieties has increased beyond 50 with the introduction of new ones from other countries such as Netherlands and Germany [5]. Most of the potatoes produced in Kenya are sold locally on the market as fresh produce and are subsequently processed into different foodstuffs either at the household or at industrial level. Furthermore, processed potato products such as crisps and French fries (locally known as chips) are on high demand among urban consumers and hence are a great part of menus in restaurants and hotels in major urban centers [6]. This, therefore, makes the potato a major part of the diet of many Kenyan consumers.

Postharvest management of the potato is an important factor not only in preventing postharvest losses but also in maintaining its nutritional quality. Furthermore, the safety of the potato for consumption is greatly influenced by postharvest management and storage. This is because the potato contains glycoalkaloids (GAs), a family of steroidal toxic secondary metabolites that occur in all parts of the potato as natural toxins, synthesized as a form of defense against parasites and diseases due to their antimicrobial, insecticidal, and fungicidal properties [7]. Of interest to food safety is the fact that these toxins can be harmful to humans if consumed in large quantities [8]. Toxicity can cause gastrointestinal disturbances and impaired nerve function [9, 10]. Higher doses can result in coma and even cause death [11]. The levels of these toxins are significantly affected by postharvest handling stress factors with exposure to light, storage temperatures, and injuries/bruising being important stress factors [7].

In Kenya, one of the major constraints facing the potato value chain is poor postharvest handling especially during marketing and distribution, and significant losses have been reported to occur at this stage [12]. It has been reported that, of the potatoes placed on markets in Kenya, nearly a quarter are damaged or green [12]. In Nairobi, it is not uncommon for traders to expose potatoes to unfavorable temperature, light, and other stress factors which favor the accumulation of GAs in the tubers. This does not only cause concern with respect to food losses but also to the health of potato consumers in Nairobi since these damaged or greened potatoes are sold to consumers, thus resulting in continued exposure to these lethal toxins. Addressing the aspects of postharvest handling among potato traders will help prevent food losses while at the same time promoting food safety, hence protecting consumer health. This study, therefore, sought to identify the postharvest handling practices among potato traders in open air markets in Nairobi County and their perception of the safety of greening and bruised potatoes. Results from the study will be helpful in sensitizing potato traders on the appropriate postharvest handling of potatoes in order to protect consumer health and reduce postharvest losses at the marketing stage.

## 2. Materials and Methods

### 2.1. Study Area

**Figure 1:**
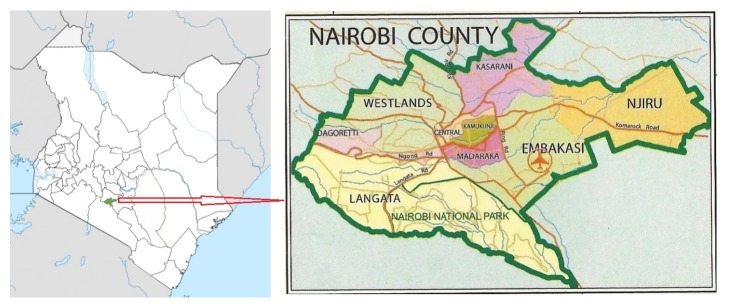
*Study area, Nairobi County.* Source: https://iddimagina.wordpress.com/tag/business-2/.

The study was carried out in Nairobi County, Kenya. The county hosts the capital city of Kenya and has nine subcounties: Makadara, Embakasi, Starehe, Langata, Kasarani, Westlands, Kamukunji, Dagorreti, and Njiru (Figure 1). The county's population is estimated to be over 3 million according to the latest census [13]. Most of the people are low income earners and hence live in slums. The county has many markets dealing in foodstuffs with most of these food markets being open air markets. Five subcounties were purposively selected for this study: Dagoretti, Westlands, Embakasi, Kamukunji, and Starehe. These were purposively selected because most of Nairobi's population is concentrated in these areas. From these 5 subcounties, 5 major markets were purposively selected from which traders were interviewed. The 5 markets were Kawangware (Dagoretti), Kangemi (Westlands), Wakulima (Starehe), Kona (Embakasi), and Gikomba (Kamukunji).

### 2.2. Study Design

The study was of a cross-sectional study design employing qualitative data collection methods through interviews and observation. The study population consisted of open air market traders who sell potato tubers. A randomly chosen sample of 100 traders, obtained using the Fischer's formula [14], participated in the study. A semistructured questionnaire was used for this study. The questionnaire was written in English and captured information on demographic characteristics and postharvest handling practices of potatoes such as transportation, exposure to sunlight, storage, action taken for greened and injured/bruised potatoes, and any other relevant information. Consent to participate in the study was sought from the respondents where a consent form was signed. Before the survey, the questionnaire was pretested on a randomly picked sample of 20 traders (with similar characteristics as the study sample) from Kawangware and Kangemi markets. This was important in ensuring that the questions will be understood by the respondents and to estimate the time that will be taken to complete the questionnaire.

### 2.3. Data Analysis

Data obtained were coded and entered into SPSS for Windows software (IBM version 21) and analyzed. Descriptive statistics, namely, percentages and frequencies, were used to express the results of sociodemographic characteristics of the study population and the different postharvest handling practices. Chi-square test of significance was used to test for any existing significant associations between the various variables under study with a p-value = 0.05 being set as the level of significance.

## 3. Results and Discussion

### 3.1. Sociodemographic Characteristics of the Respondents

The sociodemographic characteristics of the potato traders are represented in Table 1. More than half of the traders were male (53%). The fact that there were more male traders than female traders concurs with Laititi (2014) who reported more male than female involvement in the potato value chain in Kenya [15]. Additionally, other research found more male traders (57%) than female traders (43%) in a study on potato traders and farmers in some of the major potato producing regions in Kenya [12]. The dominance of men over women in potato retail and trade could be explained by the fact that most of the potato farming in Kenya is of small scale and women are more involved in production activities especially at the farm level while men engage in other off-farm activities like marketing. A similar phenomenon was also observed in a study on agricultural value chains in Cameroon. Results showed slightly lower participation of women in the market especially for activities such as warehousing, transport, and wholesale. Some of the reasons given included increased household responsibilities for women; the activities on the market are sometimes physically demanding and issues such as pregnancy affect their active participation in marketing activities [16].

The age of the respondents ranged from 20 years to 80 years with the majority falling in the 30-39-year bracket (35%). With regard to education level, most of the traders had attained primary (44%) and secondary (44%) levels with only 2% of them having studied up to tertiary level.

### 3.2. Potato Varieties Traded

**Figure 2:**
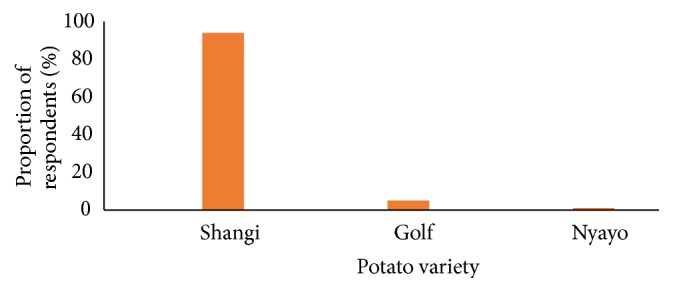
Varieties of potatoes sold in open air markets in Nairobi.

Shangi variety was the most common variety sold in Nairobi (94%) as shown in Figure 2. The other varieties identified by the study were Golf (5%) and Nyayo (1%). Previous studies have reported Shangi to be the most commonly marketed and consumed variety in Kenya [5]. The variety is common because it is early maturing and has high productivity compared to other varieties and thus is preferred by most farmers. It is also preferred by many low-income consumers due to its short cooking time, thus saving on energy costs [2]. In addition, Shangi has good cooking qualities and hence is preferred for processing of French fries, commonly known as chips [5].

**Figure 3:**
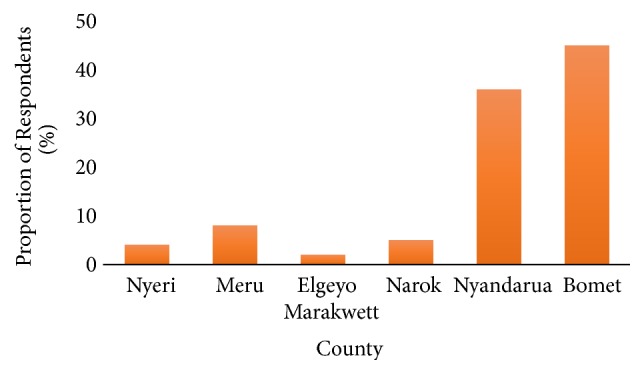
Source of ware potatoes sold in open air markets in Nairobi.

As shown in Figure 3 most of the potatoes traded in Nairobi's open air markets come from Bomet County (45%) followed by Nyandarua County (38%) with the least supply among the surveyed respondents being from Elgeyo Marakwett County (2%). Nyandarua and Bomet are some of the major potato producing regions in Kenya [4] and this could explain why most of the traders in Nairobi source their supply from these regions.

### 3.3. Postharvest Handling Practices

Ninety-three (93) traders used a vehicle (lorry or pick-up truck) or hand-pulled cart to transport their potatoes from the point of supply to the point of sale while 7 traders used motorcycles. Further assessment of the design of the back of the vehicles and hand-pulled carts used for transporting potatoes revealed that 47% had open backs while 53% had closed backs. This shows that over half of the potatoes are not exposed to sunlight during transportation since the closed backs protect the potatoes from direct sunrays. However, a sizeable proportion of potatoes (47%) are exposed to direct sunlight during transportation in vehicles with open backs. This mode of transport can be a risk factor for the development of glycoalkaloids in the tubers during transportation and subsequent storage. The mode of transport has a huge influence on development of GAs in potatoes since an open back allows for direct exposure of the tubers to unfavorable conditions such as direct sunlight and high temperatures which accelerate the development of GAs [7].

Three quarters (75%) of the potatoes on the market were left out overnight while covered with a polythene bag after the day's activities until the next day when the traders opened their businesses. The covering of potatoes with a polythene bag means that there is poor aeration and the temperatures inside the cover might rise, which is a risk factor for the development of glycoalkaloids due to exposure to high temperatures. Furthermore, since the potatoes are stored outside and sold in the open, they are exposed to sunlight, further increasing the chance of accumulation of glycoalkaloids in the tubers.

Generally, most of the potatoes take 1 to 3 days on the market, meaning that they are high moving commodities due to the high demand for potato products among Nairobi residents. However, a few of the traders who stocked large quantities of potatoes stored them for longer periods of 1 to 4 weeks. This may affect the quality of potatoes and hence a food safety risk given that potatoes are semiperishable products and longer storage periods increases the chances of greening, sprouting, and development of glycoalkaloids in the tubers, especially if stored under poor storage conditions [7].

The location, gender, and variety of potato were found to influence the postharvest handling practices of potato (Table 2). Duration taken for potatoes to reach the market was significantly (p<0.05) associated with gender, while means of transport was significantly (p<0.05) dependent on the subcounty, specific market, gender, and potato variety being traded. The means of transport preferred by traders in Starehe and Dagoretti Subcounties were vehicles which significantly (p<0.05) differed from that preferred by traders from Embakasi Subcounty where handcarts were used (Table 3). Potato traders in Kona and Gikomba markets mainly preferred vehicles as means of transport at 65.9% and 60.9%, respectively, whereas the majority (56.2%) of those in Kangemi market preferred handcarts (p<0.05). More (68.1%) males tend to use vehicles as means of transport as compared to females who only had 63% using the vehicle as a mode of transportation (p<0.05). Those transporting the golf variety of potatoes used vehicles as their means of transport.

The findings reveal different preferences on the mode of transport for different markets, locations, gender, and different potato varieties. Vehicle transportation was most preferred by significant proportions of the various segments of the population. Potato production in Kenya is mainly done in the highlands and, due to their high perishability, a faster means of transport is most preferred; however, this attracts additional costs in terms of transportation [17]. A study by Laititi in 2014 found similar results where vehicle transportation was used to deliver potatoes from various producing areas in the country to markets in Nairobi; the handcart served just as a complementary mode of transportation [15]. Therefore, the use of handcart is not the primary mode for most of potato traders in Nairobi. However, studies done in the rural areas by Kaguongo* et al*. [12] showed contrary findings with cart transportation being the most preferred means of transport due to the short distances involved.

Closed back vehicles were used for transportation mostly in Kamukunji and Starehe Subcounties at 51.3% and 65.3%, respectively, this being significantly (p<0.05) higher than other subcounties. Different vehicle designs were preferred in different markets as Kawangware and Kangemi at 55.7% and 59%, respectively, which preferred closed back modes of transport (p<0.05). The golf variety of potatoes was majorly (100%) transported using closed back vehicle designs (p<0.05).

The study findings reveal that a higher proportion of the respondents in the majority of the subcounties and markets would prefer using closed back design of vehicles to transport their potatoes. Regardless of the gender or potato variety, most of the traders would still prefer using closed back modes of transport to ferry their products. This is an effort to reduce spoilage of the potato during transportation. Further, the use of vehicles with closed backs reduces the exposure of the tubers to direct sunlight which has food safety implications as this reduces the risk of accumulation of glycoalkaloids in the potatoes.

### 3.4. Exposure of Potatoes to Sunlight

From observations, it was noted that more than half (69%) of the potatoes in the surveyed markets were exposed to sunlight. Potato variety and gender were found to be factors that predicted likelihood of exposure of potato to sunlight as shown in Table 4. Male traders were five times more likely to expose the potatoes to sunlight than the female traders. The logit regression model for the equation is as follows.

*y* = *Ax* + *Bz*, where y is exposure of potato to sunlight,* x *and *z* are the independent variables, gender and potato variety, and A and B are constants.* A *and *B* accounted for 30% change in* y*.

The findings show that exposure of potatoes to sunlight is a common practice among the traders regardless of education level, age, and all locations with no difference in handling to curtail the same. Individuals who were males were five times more likely to have their potatoes exposed to light than the females. Purchase of potatoes from the male traders poses greater risk of exposure to consumption of glycoalkaloids. Interestingly, the education level of the traders did not influence how they handled the potatoes with respect to exposure to sunlight.

### 3.5. Trader Perception of the Safety of Greened, Bruised, or Sprouting Potatoes

**Figure 4:**
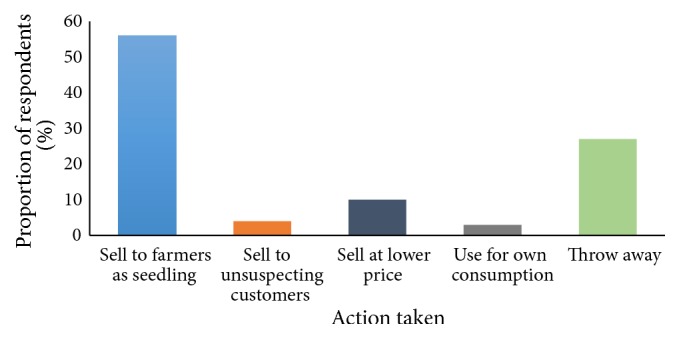
Action taken by traders for potatoes that show signs of greening.

More than half (56%) of potato traders in open air markets in Nairobi sell greened potatoes or potatoes that show signs of greening to farmers as seed (Figure 4). This shows that almost half of the greened potatoes on the market are sold back to the farmers for replanting. This observation supports earlier findings where poor access to certified/good quality seed was found to be one of the major constraints by potato farmers in Kenya [2]. However, some of these greening potatoes are sold to consumers at a lower price (20%) or some dubious traders stash them among fresh potatoes and sell them to unsuspecting customers (4%). This may pose a serious health risk to consumers if these potatoes that show signs of greening are consumed since greening in potatoes is a phenomenon associated with the formation of glycoalkaloids. A sizeable number of traders (27%) threw away potatoes that showed signs of greening. This means that potato losses are one of the major challenges experienced by traders, which translates to major food losses in a country that is already grappling with food insecurity. A study by Kaguongo* et al*. [12] found that postharvest loss is a major problem facing many potato farmers in Kenya with 19% of produce being lost per season, translating to 815,000 tonnes lost which represents an estimated monetary loss of Ksh. 12.9 billion every year [12]. This problem can be mitigated through efforts such as sensitizing the potato value chain players on proper storage, development of modern and affordable storage technologies, and development of potato varieties with longer storage duration.

**Figure 5:**
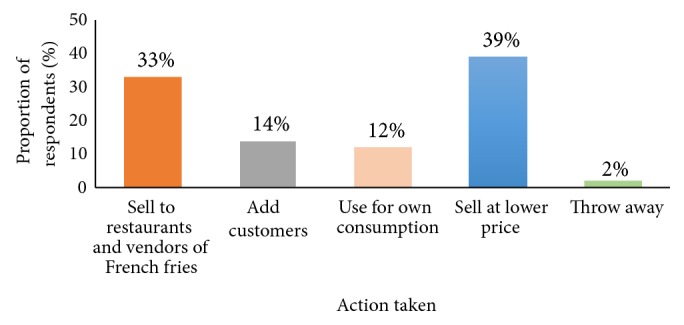
Action taken by traders on bruised or sprouting potatoes.

Figure 5 shows various actions taken by potato traders for potatoes that are bruised or sprout while still on the market. Bruising and sprouting are some of the factors that contribute to increased levels of glycoalkaloids in potatoes [7]; hence potatoes that are sprouting or have been bruised should not be consumed. Only a small number of traders (2%) threw away such potatoes. The remaining traders either sold bruised or sprouting potatoes directly to consumers at a lower price or sold them to restaurants or other vendors who traded in potato products especially French fries.

The present study sought to assess the knowledge of traders regarding the harmful effects of consuming greening or sprouting potatoes. More than half of the traders (53%) said that consuming greening or sprouting potatoes does not have any harmful effects on health; 28% said that greening or sprouting potatoes are harmful to health, while 19% did not know whether greened or sprouting potatoes were harmful to health or not. This means that a large number of them do not know the harmful effects of consuming such potatoes which may explain why some of them sold such potatoes to unsuspecting customers, sold them at a lower price, used them for their own consumption, or added them to their customers as an incentive.

Sociodemographic characteristics of the traders significantly (p<0.05) influenced their perception of the safety of potatoes (Table 5). Majority of the traders from Dagoretti and Westlands Subcounties sold greening potatoes as seed at 68.5% and 72.2%, respectively, a higher proportion than other subcounties (p<0.05). Traders who were older or were from Kawangware and Kangemi markets would also prefer to sell greening potatoes as seed. Damaged or bruised potatoes are majorly sold at discounted prices by the majority of the traders who are males, from Dagoretti Subcounty or Kawangware markets; this shows that potato buyers in these regions may be more exposed to potato glycoalkaloids than the other regions/markets.

The findings show gender, location, and age as the possible factors that may influence trader perception of the safety of potatoes. Best practice of avoiding consumption of greening potatoes was mainly among traders in Kawangware and Kangemi markets, Dagoretti and Westlands Subcounties, or those of older ages. However, poor food safety practices of selling damaged or bruised potatoes were common among male traders and in Dagoretti Subcounty and Kawangware market. Stress such as bruising and damage results in increased glycoalkaloid levels in potatoes [18]. Inasmuch as these potatoes are unsafe, the profit incentive is used by traders to avoid losses on their end.

The variety of ware potato was significantly (p<0.05) associated with the traders' understanding of greening in potatoes. Majority of traders that traded in Shangi variety perceived greening in potatoes as a sign of the potato becoming a seed, whereas those trading in Golf variety perceived it as a sign of spoilage. Shangi variety is the most utilized variety in Kenya; thus most traders would prefer not to suffer any losses by discarding greening potatoes and would rather sell them off as seed.

## 4. Conclusion and Recommendations

Postharvest handling practices among the potato traders in open air markets in Nairobi may impact negatively the quality of the potatoes especially in terms of safety. There is a low level of knowledge on the health effects of consuming greened, bruised, or sprouting potatoes leading to the poor perception of potato safety among the traders. More than half of the potatoes on the market are exposed to unfavorable temperature and light conditions as seen through the direct exposure of the tubers to sunlight. This means potato consumers in Nairobi may be experiencing long term exposure to these toxins due to buying of greening, bruised, or sprouting potatoes for home consumption or consuming of potato products such as French fries from restaurants or roadside vendors who use greening, bruised, or sprouting potatoes sold to them by the traders to make these potato products.

Therefore, it is important that all potato traders be continuously sensitized and educated on the health effects of glycoalkaloids and proper postharvest handling of potatoes to prevent continued consumer exposure to these toxins. Farmers and transporters of the tubers to the market should also be included in the sensitization activities. In addition, the Nairobi County Government should channel resources towards building permanent stalls that have proper storage places with proper protection against the sun to prevent against direct exposure of potatoes to the sun. The traders should also be facilitated to acquire sacks and bags made from other materials such as sisal or net bags which allow for proper aeration and less build-up of heat to store their potatoes.

## Figures and Tables

**Table 1:** Sociodemographic characteristics of potato traders in open air markets in Nairobi County.

Characteristic	Frequency (n=100)	Percentage (%)
*Gender*		
Male	53	53
Female	47	47
*Age*		
20-29 years	20	20
30-39 years	36	36
40-49 years	20	20
50-59 years	18	18
>59 years	6	6
* Education Level*		
No education	10	10
Primary	44	44
Secondary	44	44
Tertiary	2	2

**Table 2:** Association between postharvest handling practices and socioeconomic and demographic characteristics.

Postharvest handling practices	Subcounty	Market	Gender	Level of education	Potato variety	Age
Duration taken to market	5.18	4.23	8.80*∗*	3.21	2.20	7.51
Means of transport	38.55*∗*	38.89*∗*	9.62*∗*	5.34	7.25*∗*	9.90
Vehicle/handcart design	32.89*∗*	32.89*∗*	9.36*∗*	6.78	9.36*∗*	5.49
Storage of potato	8.33	8.33	2.27	2.25	1.14	3.31
Duration of storage of potato	10.98	10.98	8.84	8.14	8.58	8.54

*∗*Significant at p<0.05.

**Table 3:** Preferences (%) for different modes of transport.

Factors		Means of transport		Design of the vehicle	
		Vehicle	Handcart	Open back	Closed back
Subcounty	Dagoretti	84.4	15.6	49.6	50.4
	Westlands	66.7	33.3	57.1	42.9
	Kamukunji	52.6	47.4	48.7	51.3
	Starehe	79.2	20.8	34.7	65.3
	Embakasi	42.2	57.8	59.2	40.8
Market	Kawangware	49.2	50.8	44.3	55.7
	Kangemi	43.8	56.2	41	59
	Gikomba	60.9	39.1	56.2	43.8
	Wakulima	56.7	43.3	67.1	32.9
	Kona	65.9	34.1	49.9	50.1
Gender	Male	68.1	31.9	50	50
	Female	63	37	49.4	50.6
Potato variety	Shangi	62.8	37.2	46.5	53.5
	Golf	100	0	0	100

**Table 4:** Logit model for exposure of potatoes to sunlight.

Variable		Odds Ratio
Subcounty	Dagoretti	0.638
	Westlands	0.542
	Kamukunji	1.486
	Starehe	1.791
	Embakasi^R^	
Gender	Male	5.256*∗*
	Female^R^	
Education level	Primary	0.431
	Secondary	1.008
	Tertiary	0.694
	No education^R^	
Variety of potato	Shangi	0.060*∗*
	Golf^R^	
Supply of potatoes	Own production	0.298
	Buy from other retailers^R^	
Age		2.547

*∗*Significant at p<0.05, R-reference category, R^2^=0.30, constant=0.00.

**Table 5:** Factors associated with trader perception of safety of potatoes.

Traders' understanding of greening in potatoes (%)							

Socioeconomic and demographic factors		Seed	Effects of light or high temperature	Effect of plant disease	Immature potato	Spoilage	P-value

Potato variety	Shangi	51.6	25.8	0	4.3	18.3	0.04
	Golf	14.3	28.6	14.3	0	42.9	

Actions taken by traders for potatoes that show greening							

		Sell as seed	Sell to consumers	Sell at lower price	Consume it	Dispose	P-value

Subcounty	Dagoretti	68.5	5.1	15.7	0	10.7	0.02
	Westlands	72.2	0	11.2	0	16.6	
	Kamukunji	58.1	10.6	5.1	5.1	21.2	
	Starehe	34.7	0	0	4.2	61.1	
	Embakasi	53.1	5.6	17.5	0	23.7	

Actions taken by traders for potatoes that show greening							

Socioeconomic and demographic factors		Sell as seed	Sell to consumers	Sell at lower price	Consume it	Dispose	P-value

Market	Kawangware	68.5	5.1	15.7	0	10.7	0.02
	Kangemi	72.2	0	11.2	0	16.6	
	Gikomba	58.1	10.6	5.1	5.1	21.2	
	Wakulima	34.7	0	0	4.2	61.1	
	Kona	53.1	5.6	17.5	0	23.7	
Age	<20	0	100	0	0	0	0.02
	20-25	36	11.9	16.1	0	36	
	26-30	71.4	0	5.8	2.7	20.1	
	>30	57.1	0	8.5	2.7	31.6	

Actions taken by traders for potatoes that show signs of damage/bruising							

Socioeconomic and demographic factors		Sell to vendors or restaurants	Given as additional items to customers	Consume	Sell at discounted price	Dispose	P-value

Subcounty	Dagoretti	20.9	20.9	5.3	52.9	0	0.00
	Westlands	15.9	15.9	20.8	47.3	0	
	Kamukunji	31.6	10.7	20.9	31.6	5.3	
	Starehe	56	12.6	12.6	12.6	6.3	
	Embakasi	42	10.6	0	47.3	0	
Market	Kawangware	20.9	20.9	5.3	52.9	0	0.00
	Kangemi	15.9	15.9	20.8	47.3	0	
	Gikomba	31.6	10.7	20.9	31.6	5.3	
	Wakulima	56	12.6	12.6	12.6	6.3	
	Kona	42	10.6	0	47.3	0	
Gender	Male	34.8	17.4	2.2	43.4	2.2	0.00
	Female	30.4	10.8	21.8	34.8	2.2	

## Data Availability

The data used to support the findings of this study are available from the corresponding author upon request.
